# Solving Large-Scale Inverse Magnetostatic Problems using the Adjoint Method

**DOI:** 10.1038/srep40816

**Published:** 2017-01-18

**Authors:** Florian Bruckner, Claas Abert, Gregor Wautischer, Christian Huber, Christoph Vogler, Michael Hinze, Dieter Suess

**Affiliations:** 1 Christian Doppler Laboratory of Advanced Magnetic Sensing and Materials, Institute of Solid State Physics, Vienna University of Technology, Austria; 2 Institute of Solid State Physics, Vienna University of Technology, Austria; 3 Department of Mathematics, University of Hamburg, Germany

## Abstract

An efficient algorithm for the reconstruction of the magnetization state within magnetic components is presented. The occurring inverse magnetostatic problem is solved by means of an adjoint approach, based on the Fredkin-Koehler method for the solution of the forward problem. Due to the use of hybrid FEM-BEM coupling combined with matrix compression techniques the resulting algorithm is well suited for large-scale problems. Furthermore the reconstruction of the magnetization state within a permanent magnet as well as an optimal design application are demonstrated.

Magnetic materials are used in a wide range of applications, ranging from permanent magnets, magnetic machines, up to magnetic sensors and magnetic recording devices. Solving the Inverse Magnetostatic Problem allows to reconstruct the internal magnetization state of a magnetic component, by means of magnetic field measurements outside of the magnetic part, which is of importance for quality control. Compared with the forward problem, where the magnetic state is known and the strayfield is calculated, inverse problems are much harder to solve, since they typically are much worse conditioned and often not uniquely solveable. Inverse problem solvers are based on stable and reliable solvers for the forward problem. In the case of magnetostatic Maxwell equations, Finite Element (FEM) formulations, combined with methods to handle the open-boundary, have proven to be the methods of choice for many efficient and accurate methods^1^,^2^,^3^.

The applications of inverse problems can be coarsely divided into optimal design and source identification. Optimal design problems define a desired strayfield and try to calculate optimal material distributions or geometries to reach these requirements as accurate as possible^4^,^5^. In contrast to this, source identification problems try to reconstruct the state of existing magnetic components. The identification of (permanent) magnetic materials has e.g. been successfully applied for reconstructing the state of magnetic rollers used in copy machines^6^, magnetically biased chokes^7^, or even for the magnetic anomaly created by ferromagnetic ships^8^.

The presented solver for the inverse 3D magnetostatic Maxwell equations, is based on the well-established Fredkin-Koehler-Method^3^, which uses a hybrid FEM-BEM coupling for efficient handling of the open-boundary conditions. Combined with a hierarchical matrix compression technique for the dense boundary integral matrices, the algorithm is able to handle large-scale problems. Additionally, the use of a general Tikhonov regularization (see e.g. ref. 9), provides a very flexible means to define application specific regularizations.

## Adjoint Method

### Forward Problem

The demagnetization field of a magnetic body is defined as 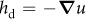, where the magnetic scalar potential *u* is given by


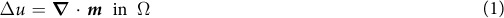



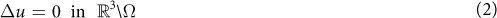


with jump and boundary conditions


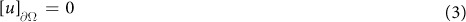



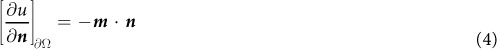



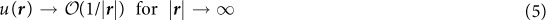


where ***m*** is the magnetization and 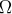 is the magnetic region.

**Figure 1:**
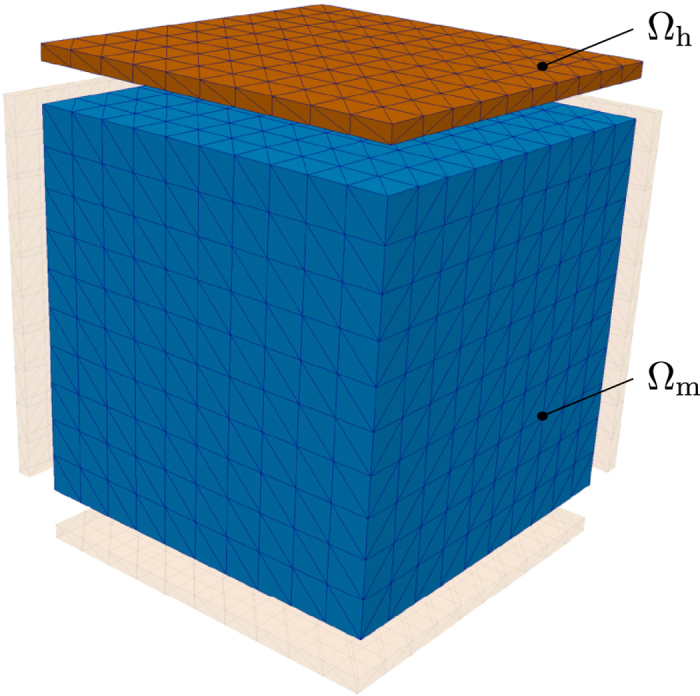
Discretized magnetization region 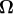
_m_ (blue) and measurement region 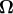
_h_ (brown). Since the strayfield problem is scale-invariant, length units are omitted. The magnetization is defined on a unit cube. Measurement boxes of thickness 0.04 are located next to each side of the cube, using an airgap of 0.1 (the 2 boxes in front are not shown in the figure).

The forward problem requires the solution of the potential *u* on the region 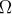
_h_ generated by the magnetization in a magnetic region 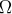
_m_ (see Fig. 1). This problem is solved by considering a single region 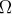 = 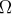
_m_ ∪ 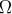
_h_ with ***m***(***x***) = 0 for ***x*** ∈ 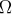
_h_. The hybrid FEM/BEM method introduced by Fredkin and Koehler^3^ is one of the most accurate methods for the solution of this problem and will be used in the following. Consider the following splitting of the solution *u*:


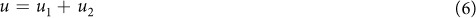


Here *u*_1_ is defined by


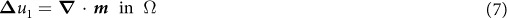



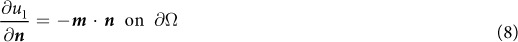



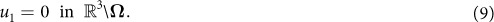


This Neumann problem is solved with the finite-element method. While *u*_1_ solves for the right-hand side ***m*** and fulfills the jump condition of the normal derivative −***m*** · ***n***, it is not continuous across ∂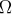 as required. This jump is compensated by *u*_2_ which is defined as


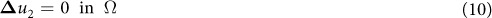



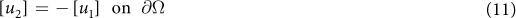



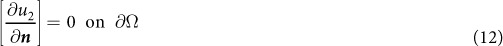



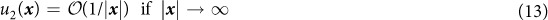


This system is solved by the following double-layer potential


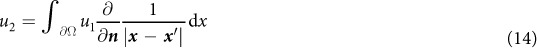


For efficiency reasons the double-layer potential is only computed on the boundary ∂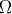 using a Galerkin boundary-element method. Subsequently these values are used as Dirichlet boundary conditions to solve *u*_2_ within 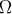 using the finite-element method.

All potentials are calculated using piecewise linear basis function (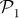) and the derived strayfield would be constant within each element. Thus, a mass lumping procedure needs to be used to project the field onto piecewise linear basis functions which are defined on each vertex of the mesh.

### Inverse Problem

The inverse problem can be understood as a PDE constrained optimization problem. Due to the ill-posedness of the inverse problem, some additional information needs to be provided to allow reasonable results. This can be achieved by using Tikhonov regularization, which uses an additional penalty term which should be considered for the optimization. A possible candidate for the objective function is


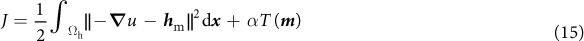


where ***h***_m_ is the prescribed (measured) field in 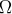
_h_ and *α* is the Tikhonov constant corresponding to the regularization functional *T*(***m***). This functional should be minimized, constrained by


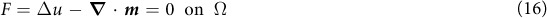


with boundary conditions as given above. We aim to solve this problem using a gradient based iterative minimizer. The constraint *F* gives an implicit expression for *u*(***m***) which allows to directly calculate the desired gradient by


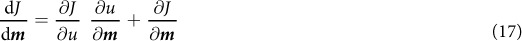


The inefficient direct calculation of the term 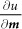 can be avoided using the adjoint approach, which makes use of the derivative of the constraint equation to eliminate the problematic term. Compared with a naive implementation using a dense system matrix 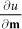, the computational as well as storage costs can be reduced from 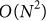 to 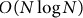:


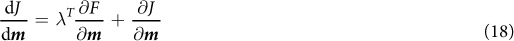


where *λ* is given by the adjoint equation


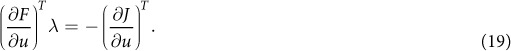


Since the Poisson problem is self-adjoint, the adjoint system (19) can be solved along the lines of the forward problem. Computing the variational derivative on the RHS yields


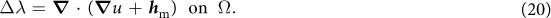


where the sources (RHS) live on 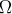
_h_ and the solution is only computed on 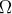
_m_. The same boundary conditions as for the forward problem hold. Thus, the above described hybrid method is applied. The gradient of *J* is then finally given by


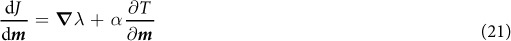


Note that 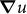 and 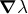 are projected onto 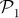 before computing (20) and (21) respectively. The algorithm is implemented using Magnum.Fe^10^, which is based on the finite element library FEniCS^11^. This allows a very comfortable definition of the regularization functional. Furthermore, automatic differentiation can be used for the calculation of the corresponding partial derivatives. The algorithm was verified by comparison with a FEM-only implementation, using the dolfin-adjoint library^12^, which allows to automatically derive the adjoint equation for a given forward problem.

## Numerical Experiments

The presented algorithm is validated by the reconstruction of the flower-state within a magnetic unit cube. The strayfield is calculated within measurement planes next to each side of the cube (see Fig. 1). The magnetic state of the cube is parametrized by


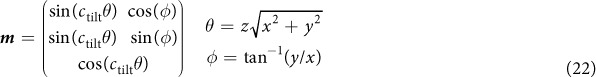


where 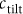 is an open parameter that allows to change the strength of the flower state. For the proper reconstruction of the magnetic state additional knowledge about the solution needs to be provided. For all presented results a smooth reconstructed magnetization is desired which suggests using the following default regularization functional


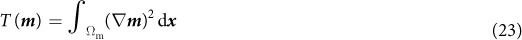


For this specific flower-state the absolute value of the magnetization is known to be constant. Thus, the solution of the inverse problem could be simplified by using the following penaltization functional


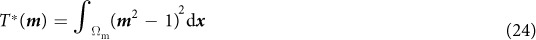


The assumption of a constant magnetization may be a good approximation for (isotropic) permanent magnetic materials. Due to the large magnetic remanence and the relatively small susceptibility the induced magnetization may be negligible (see ref. 13 for a simple model of isotropic permanent magnetic materials).

**Figure 2:**
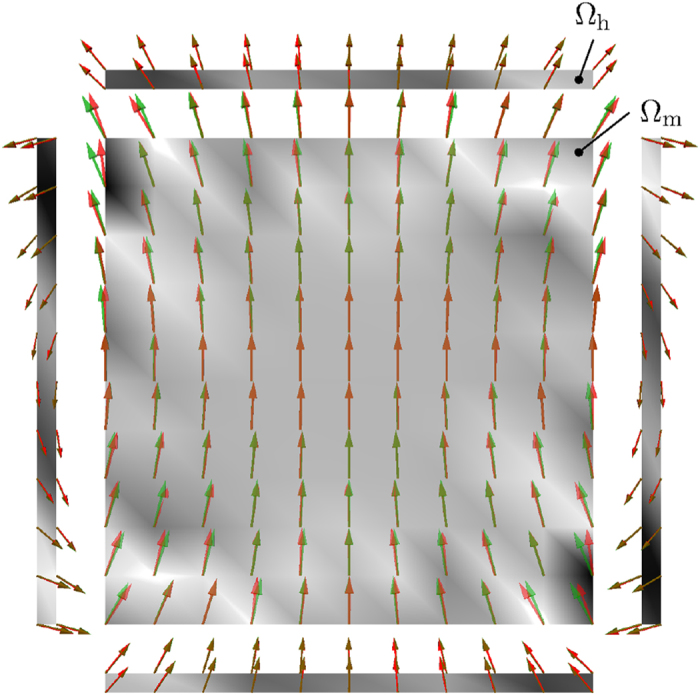
Reconstruction of a flower-state within a unit cube according to Eqn. (22) using *c*_tilt_ = 2. A cut through the *y* = 0 plane is visualized. Starting from the initial parametrized flower state in 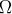
_*m*_ the magnetic strayfield is calculated within the fieldboxes 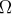
_*h*_ (green arrows). In order to simulate measurement errors a Gaussian noise with σ = 10^−4^ has been added to the forward strayfield. The reconstructed magnetization as well as strayfield are computed using *α* = 10^−3^ (red arrows). The relative differences of initial and reconstructed states are indicated by the gray-scale. Maximal relative errors of the *x*-components amount to 0.25 for the magnetization, and 5 · 10^−3^ for the induced magnetic field, respectively.

A Gaussian noise with zero mean and a standard deviation σ = 10^−4^ has been added to the field, calculated by the forward problem, which should simulate unavoidable measurement errors. The minimization problem is solved by a gradient descent method combined with a line-search strategy. As expected, reconstruction without using a proper regularization leads to large magnetization vectors near to the edges of the unit cube. Increasing the regularization parameter *α*, first avoids the over-fitting of the noisy measurement data, but finally leads to blurring of the reconstruction results if *α* gets too large. Determining the optimal alpha is a crucial step for the solution of an inverse problem. The results for the reconstruction of a flower state with *c*_tilt_ = 2 using *α* = 10^−3^ is visualized in Fig. 2. Although there are some deviations of the reconstructed magnetization from the reference state. It can be seen that the created strayfield is nearly identical. As stated above this is a clear indication of the bad condition of the inverse problem.

**Figure 3:**
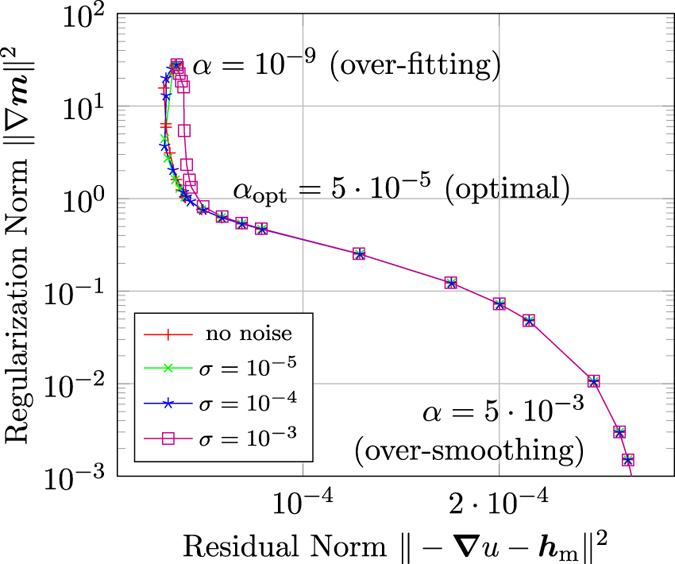
L-curves for the reconstruction of the flower state for different noise levels *σ*.

Using the so-called L-curve method^14^, allows to visualize the trade-off between the reconstruction of the strayfield and the fulfillment of the regularization constraint. Plotting the regularization norm (also called solution norm) 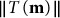 over the residual norm 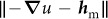 for different regularization parameters *α*, shows an L-shaped curve. The optimal *α*_opt_ can be selected at the corner of the L-curve which means that *α* is large enough to reduce the regularization norm significantly, but it does not change the residual norm too much. The resulting L-curves for the reconstruction of the flower state for different noise levels are summarized in Fig. 3. An optimal value *α*_opt_ ≈ 5 · 10^−5^ can be found.

The application of the presented method to optimal design problems should now be demonstrated by means of a Halbach cylinder configuration. The goal is to find a magnetization configuration within a cylindrical domain, which creates a homogeneous strayfield inside of the cylinder. The magnetization domain 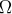
_m_ has an outer radius *r*_*o*_ = 1.0, an inner radius *r*_*i*_ = 0.6, and a height of *h* = 2.0, while a cylindrical measurement domain 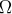
_h_ with radius *r*_*m*_ = 0.5 and the same height is used. The magnetization vectors should have constant norm, which suggests using the constant-norm penaltization functional (24). The analytic solution for a cylindical Halbach array^15^ can be expressed in cylindrical coordinates as


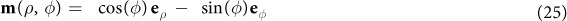


where *ρ, ϕ* are the cylindrical coordinates, with the corresponding unit vectors **e**_*ρ*_, **e**_*ϕ*_.

**Figure 4:**
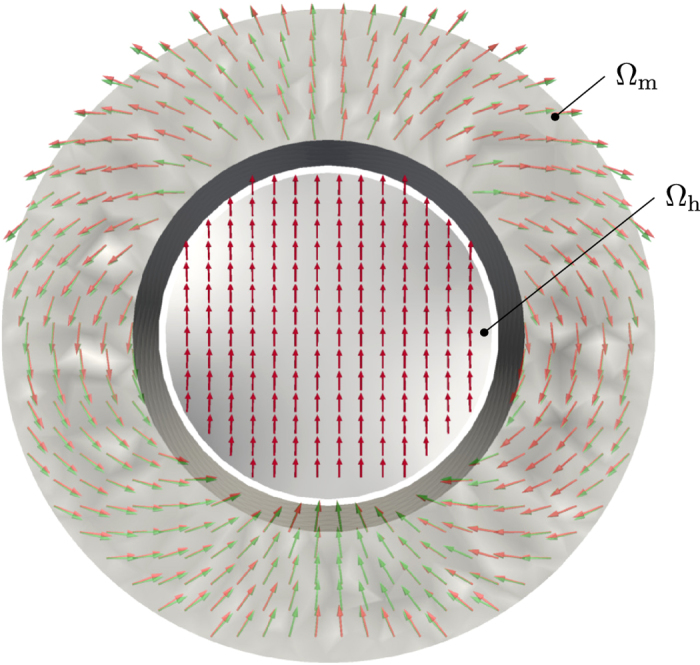
Optimal design problem of a Halbach cylinder creating a homogeneous strayfield inside of the cylinder. Starting from a homogeneous strayfield the presented algorithm reproduces a Halbach like magnetization configuration within 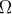
_m_ (red arrows). A constant-norm regularization with *α* = 10^4^ is used and shows a nearly perfect match with the analytical solution (green arrows). The resulting strayfield is calculated inside 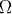
_h_ and shows a nearly homogeneous distribution (red arrows). The relative errors of the magnetization magnitude and the reconstruced strayfield are indicated by the gray-scale, and their maximum amount to 2% and 6%, respectively.

As visualized in Fig. 4 there is a nearly perfect match of the reconstructed and the analytical Halbach configuration.

## Conclusion

An efficient algorithm for the solution of inverse problems has been introduced. The use of the Finite Element library FEniCS allows to easily define application specific regularization functionals in a very flexible way. Thus, the implemented algorithm is suitable for a wide range of applications including reverse engineering of magnetic components, design and optimization of magnetic circuits and topology optimization, respectively. Using the hybrid FEM-BEM method proposed by Fredkin-Koehler allows to handle the open-boundary problem accurately and without the need for global mesh including a large airbox. Source identification has been validated by the successful reconstruction of the magnetic flower-state within a unit cube by means of Tikhonov regularization. The selection of a suitable regularization parameter has been demonstrated using the L-curve method. Finally, the application of the method to an optimal design problem has been demonstrated by means of an Halbach cylinder, which is nearly perfectly reproduced.

## Additional Information

**How to cite this article:** Bruckner, F. *et al*. Solving Large-Scale Inverse Magnetostatic Problems using the Adjoint Method. *Sci. Rep.* **7**, 40816; doi: 10.1038/srep40816 (2017).

**Publisher's note:** Springer Nature remains neutral with regard to jurisdictional claims in published maps and institutional affiliations.

## References

[b1] Brunotte X., Meunier G. & Imhoff J. Finite element modeling of unbounded problems using transformations: A rigorous, powerful and easy solution. IEEE Trans. Magn. 28, 1663–1666 (1992).

[b2] Khebir A., Kouki A. & Mittra R. Asymptotic Boundary Conditions for Finite Element Analysis of Three-Dimensional Transmission Line Discontinuities. IEEE Trans. Microwave Theory Tech. 38, 1427–1432 (1990).

[b3] Fredkin D. & Koehler T. Hybrid method for computing demagnetizing fields. IEEE Trans. Magn. 26, 415–417 (1990).

[b4] Neittaanmki P., Rudnicki M. & Savini A. Inverse Problems and Optimal Design in Electricity and Magnetism (Clarendon Press, 1996).

[b5] Yoo J. & Kikuchi N. Topology optimization in magnetic fields using the homogenization design method. Int. J. Numer. Methods Eng. 48, 1463–1479 (2000).

[b6] Igarashi H., Honma T. & Kost A. Inverse inference of magnetization distribution in cylindrical permanent magnets. IEEE Trans. Magn. 36, 1168–1171 (2000).

[b7] Husstedt H. & Kaltenbacher M. Detailed analysis of permanent magnets by means of free field measurements. J. Appl. Phys. 115, 17E302 (2014).

[b8] Chadebec O., Coulomb J. L., Bongiraud J. P., Cauffet G. & Thiec P. L. Recent improvements for solving inverse magnetostatic problem applied to thin shells. IEEE Trans. Magn. 38, 1005–1008 (2002).

[b9] Calvetti D., Morigi S., Reichel L. & Sgallari F. Tikhonov regularization and the L-curve for large discrete ill-posed problems. J. Comput. Appl. Math. 123, 423–446 (2000).

[b10] Abert C., Exl L., Bruckner F., Drews A. & Suess D. magnum.fe: A micromagnetic finite-element simulation code based on FEniCS. J. Magn. Magn. Mater. 345, 29–35 (2013).

[b11] Logg A., Mardal K.-A. & Wells G. Automated Solution of Differential Equations by the Finite Element Method: The FEniCS Book (Springer Science & Business Media, 2012).

[b12] Farrell P. E., Ham D. A., Funke S. W. & Rognes M. E. Automated Derivation of the Adjoint of High-Level Transient Finite Element Programs. SIAM J. Sci. Comput. 35, C369–C393 (2013).

[b13] Bruckner F. . Macroscopic simulation of isotropic permanent magnets. J. Magn. Magn. Mater. 401, 875–879 (2016).

[b14] Hansen P. C. & OLeary D. P. The Use of the L-Curve in the Regularization of Discrete Ill-Posed Problems. SIAM J. Sci. Comput. 14, 1487–1503 (1993).

[b15] Halbach K. Design of permanent multipole magnets with oriented rare earth cobalt material. Nucl. Instrum. Methods 169, 1–10 (1980).

